# Ovarian Neoplasm: Delivering Suspicion of Cancer in the Emergency Department

**DOI:** 10.7759/cureus.22738

**Published:** 2022-03-01

**Authors:** Mfoniso Ekpo, Laura D Bauler, Kathryn Redinger

**Affiliations:** 1 Emergency Medicine, Western Michigan University Homer Stryker M.D. School of Medicine, Kalamazoo, USA; 2 Biomedical Sciences, Western Michigan University Homer Stryker M.D. School of Medicine, Kalamazoo, USA

**Keywords:** patient follow-up, presumptive diagnosis, ovarian cyst, patient provider communication, cancer diagnosis

## Abstract

Cancer is not infrequently detected in the Emergency Department (ED) and is sometimes even an incidental finding on imaging. Since the ED is designed to identify and treat acutely ill patients, the time providers can spend with patients and the depth of investigation into patient conditions is limited. However, Emergency Medicine physicians must ensure the appropriate follow-up for patients with presumptive diagnosis of cancer to ensure timely confirmatory testing, prompt treatment, and accurate prognosis. A 26-year-old woman presented to the ED for evaluation of abdominal pain and urinary complaints and was ultimately found to have a 36cm ovarian mass that was suspicious for neoplasm. The mass caused obstruction of urinary outflow leading the patient to develop a urinary tract infection. Emergency Medicine physicians are faced with the challenge of having limited time and short-lived doctor-patient relationships. In cases of suspicious findings, balancing the urgency of follow-up without causing undue harm from heightened anxiety for patients is essential. It is important to discuss findings that may be concerning for cancer with both clear verbal and written communication. Employ strategies such as direct communication with primary care physicians and outpatient specialists via phone consultation and electronic medical record messaging, as well as providing clear discharge instructions in-person and in-writing to the patient including whom to call and the time frame for follow-up.

## Introduction

The Emergency Department (ED) functions as a relay station for both emergent and non-emergent cases in the hospital, entry into the hospital system, and a source of continuing care seeing millions of patients every year [1,2]. The role of an ED physician is to prevent and reduce mortality in time-sensitive emergency cases [3]. Definitive diagnoses are often elusive in the ED, therefore when a patient presents with symptoms, laboratory tests, or radiographic imaging consistent with cancer, confirmatory testing with biopsy, tumor markers, and subsequent treatment decisions fall outside the scope of the ED [4]. While the mere mention of cancer to the patient may be alarming, the emergency physician is tasked with delivery of a presumptive diagnosis and ensuring outpatient follow-up. The purpose of this case report is to provide tools for Emergency Medicine physicians to carefully deliver the presumptive diagnosis of cancer in the ED.

## Case presentation

A 26-year-old otherwise healthy, nulligravida female presented to the ED with worsening right lower quadrant pain, diarrhea, nausea, and vomiting for four days. She also had low back discomfort and abdominal discomfort which she thought was secondary to forceful vomiting. She had taken a muscle relaxant earlier that morning but the pain in her right lower quadrant persisted. Her primary care physician (PCP) advised her earlier in the day that she may have a kidney stone and should go to the ED for evaluation. Over the past two years, she had experienced recurrent and increasing abdominal discomfort, increased heartburn, right and left lower quadrant pain with back pain, and fluctuating periods of diarrhea and constipation. Of note, she was seen the same ED 12 months prior and was found to have a urinary tract infection and was treated with antibiotics. Over the past 12 months she had followed up with her PCP and various urgent care clinics approximately four times with similar symptoms; no imaging had been performed at any of those visits. Her history was significant for irregular and heavy periods since she was young. Family history was positive for pancreatic, prostate, breast, and throat cancer spanning three generations.

**Figure 1:**
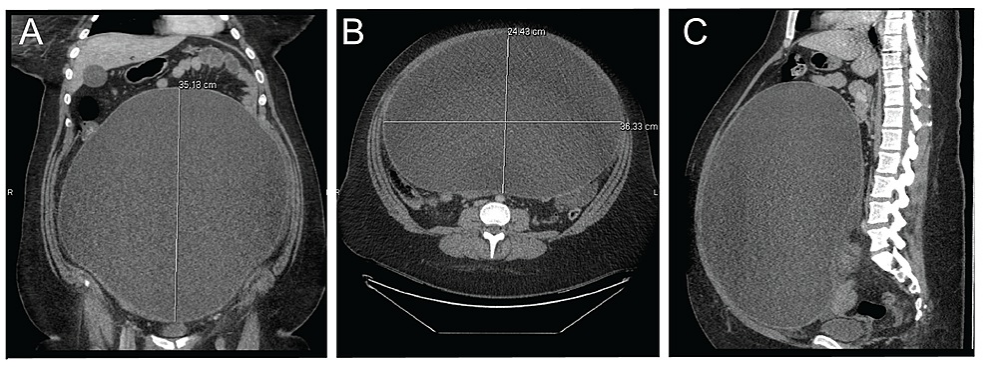
Computed Tomography Reveals an Abdominal Mass Multiplanar CT image of the abdomen and pelvis with IV contrast showing a large cystic mass extending to the pelvis and the upper abdomen which displaces bowel loops. The mass measures approximately 35 x 24 x 36 cm with a thin, peripheral rind measuring 5mm and is suspicious of a neoplasm. A: Coronal plane. B: Axial plane. C: Sagittal plane.

The patient had a noticeably tender abdominal examination but all vital signs were stable. Laboratory data revealed leukocytosis with a white blood cell count of 16.4 with a left shift, pyuria, and bacteriuria concerning for a urinary tract infection (UTI). The patient was currently being treated with trimethoprim-sulfamethoxazole for her UTI. Computed tomography (CT) scan revealed a very large cystic pelvic mass, 35x24 x36cm, extending into the abdomen (Figure 1). The mass displaced bowel loops and the colon and had a thin peripheral rind measuring 5mm. There was also free fluid in the pelvic cavity.

Due to concern for an ovarian neoplasm, the on-call surgical gynecology oncologist was consulted. An appointment was scheduled with surgical gynecology the following day, and surgery was scheduled the following week. The patient was discharged as she was tolerating fluids, showed symptom improvement of her pain, and was not showing evidence of bowel obstruction. The patient was advised to return if her symptoms worsened. She did not display evidence of metastatic disease. Further tumor marker and other genetic testing was deferred to oncology follow-up.

The patient was re-evaluated at the cancer institution six days later. The characteristics of the mass on imaging were benign, however, the patient did have an elevated CA-125 cancer marker of 107. She was diagnosed pre-operatively with a 40cm ovarian cyst, abnormal uterine bleeding, and a vulvar lesion. She underwent a minimally invasive, robotic-assisted resection of the pelvic mass. Fifteen liters of fluid was drained from the abdominopelvic cyst. Surgical pathology confirmed the abdominal mass was a large benign infarcted cyst. Histopathology revealed a unilocular cystic ovary that was smooth in appearance with no papillary outgrowth on gross and microscopic analysis. Both mucus and blood clots were present within the cyst. The final diagnosis was a benign cyst/pseudocyst without identifiable epithelial lining and absence of malignancy. Follow-up three months later found the patient well with no further complications.

## Discussion

Emergency Medicine physicians are faced with difficult conversations with patients, discussing sensitive and potentially life-altering information. The availability and use of CT imaging as a diagnostic tool has proven crucial to finding tumors, often before they become symptomatic [5]. Due to the nature of cancer diagnosis, which requires biopsy, Emergency physicians, who often encounter incidental findings of cancer on imaging, are faced with a challenging discussion with patients. These discussions require a careful balance of relaying their suspicions in a manner that incites patients to follow-up, while also not scaring patients.

Patients who present to the ED with abdominal pain, flank pain, nausea, vomiting, or a change in bowel habits are often suspected to have a urinary tract infection, kidney stones, or genitourinary disorders, as was initially the case for our patient. One study found that 51.2% of women with early-stage adnexal tumors had presenting symptoms of lower abdominal pain and pelvic pain, while 10.2% had upper abdominal or flank pain. As many as a quarter of those women were initially evaluated by EM physicians and 53.1% were found to have ovarian masses on initial imaging study using abdominal pelvic CT. Although 49% of ovarian cancer is subsequently diagnosed by an Internist, ED physicians diagnose approximately 25% of cases [6]. Ovarian cancer is rarely found in pre-menopausal women, instead functional cysts, leiomyomata, and ectopic pregnancy are the most commonly found etiologies. Post-menopausal women have an increased risk of malignancy in ovarian masses (30%) [7]. 

Bloating and a change in bowel habits are commonly reported symptoms for these patients, also observed in our patient in addition to her abdominal and flank pain [6]. Patients presenting with abdominal pain, chest pain, dyspnea, and/or headaches are often assessed via use of CT imaging by physicians as a decision-making diagnostic tool for patient care [8]. Additionally, patients with recurrent or treatment-resistant UTIs should have CT imaging completed to assess for underlying causes [9]. For detection of late-stage adnexal abnormalities, abdominopelvic CT scan is a more sensitive technique when compared to pelvic ultrasound [5,6,10]. Ovarian tumors are difficult to classify as malignant or benign using imaging analysis alone [10].

Incidental findings pose a challenge to clinical decision making. However, they are commonly found on 56.3% of abdominal CTs, 46.2% of thorax CTs, and 19.8% of head CTs. Meningiomas, pulmonary nodules, and adnexal tumors and/or cysts make up 25%, 25% and 21.4% of incidental neoplasm findings on CT [5]. While some cases may not warrant immediate care or referrals, suspicion of severe disease processes such as cancer necessitates relay of the information to the patient for follow-up. Studies indicate that 9.8% of patients had incidental findings that ED physicians deemed severe enough to disclose to patients. Patients are often advised to follow up with their PCP for subsequent repeat CTs or other diagnostic work to further characterize the findings [5].

While EDs are designed to rapidly treat emergent conditions, in times of non-emergent but important diagnoses, conveying information to patients in a way that achieves the necessary follow-up care can be challenging. For a variety of reasons including high patient volumes, limited time, frequent interruptions, and high acuity, the development of meaningful patient-provider relationships is a challenge for EM physicians. Patients may also have their own set of barriers to establishing a quick, trusting relationship [11]. Additionally, due to a reliance on rapid diagnostic tests, the EM physician cannot definitively give a cancer diagnosis but can only relay their suspicions and the need for further testing.

Patient referrals to their PCPs have a positive impact on patient care. It not only benefits the patients but can be cost-effective to the hospital [12]. The disadvantage and lack of longevity in doctor-patient relationships in the ED make it difficult to follow up with patient care. There are many factors that determine whether or not a patient will follow up with their PCP, including whether or not the patient has a PCP, their insurance, what time the patient visited the ED, when the outpatient appointment was scheduled, and the effectiveness of communication between the doctor and the patient at the time of discharge. As many as 53% of patients without a PCP who had their follow-up appointments booked on the same day of discharge had a significantly high rate of compliance for outpatient follow-up. Patients who visited the ED during weekday hours tend to follow up with their outpatient appointments, especially those who had their doctors communicate those needs and explained why the follow-up was warranted [13].

Adequate communication is essential to doctor-patient relationships, so it is important to ensure that patients are well-educated on suspicions of cancer when masses are found on CT. To improve doctor-patient communication and ensure compliance of follow-up on important but non-urgent diagnoses, doctors should discuss their findings with colleagues and encourage patient advocacy and care. Using simple language and eliminating medical jargon, providing a safe and caring environment, and providing access to further information can improve communication between provider and patients (Table 1) [14]. Relaying suspicions to the patient’s PCP will allow for appropriate care measures to be in place. The PCP can refer the patient to the right specialist and communicate with patients to ensure that their appointments are booked and attended. In cases of more severe suspicions, making referrals or even appointments at a local cancer center can help to ensure appropriate patient follow-up.

**Table 1:** Tips to Improve Communication in the ED and Ensure Patient Follow-Up

Tips	Rationale
Use simple language and explain medical jargon	The goal is to have the patient properly educated, not confused and panicked in addition to their current state of health.
	The ED is fast-paced and may be loud, impeding conversations.
	Findings should be provided in simple language, avoiding medical jargon.
	People often think cancer when they hear mass or tumor. Explain the difference between a benign and malignant tumor.
Provide a safe environment that has trust	Ensure that patients can communicate their symptoms safely while ensuring psychological safety.
	Although the ED is a fast-paced environment, be cognizant of pacing and tone, so as to not make the patient feel rushed.
	Provide emotional support.
	Give possible solutions while communicating suspicions to the patients.
Provide access to further information	Provide information about suspicions with the discharge paperwork.
	Tell patients to relay information to their PCP to ensure active listening and engagement of the patient.
	Provide ways to access the provider for clarification.

## Conclusions

Symptoms of abdominal pain, flank pain, nausea, vomiting, and change in bowel habits are symptoms that trigger a broad differential including kidney stones and urinary tract infections; ovarian masses while rare should remain a part of the differential. Imaging modalities such as CT can be used to ascertain the pathological process causing patients’ symptoms. On the occasion where ovarian masses are found on CT, it is important to communicate and educate patients on these findings. While it may be challenging in the ED to create long-lasting doctor-patient relationships, appropriate education and communication of life-changing, yet non-emergent suspicions of cancer can ensure that patients receive the necessary follow-up care.
